# Ultra-high-performance core–shell structured Ru@Pt/C catalyst prepared by a facile pulse electrochemical deposition method

**DOI:** 10.1038/srep11604

**Published:** 2015-08-03

**Authors:** Dan Chen, Yuexia Li, Shijun Liao, Dong Su, Huiyu Song, Yingwei Li, Lijun Yang, Can Li

**Affiliations:** 1 Key Laboratory of Fuel Cell Technology of Guangdong Province, School of Chemistry and Chemical Engineering, South China University of Technology, Guangzhou, 510641, China; 2 Department of Chemistry, Datong University, Datong, 037009, China; 3 Functional Nanomaterials Center, Brookhaven National Laboratory, Upton, NY 11973, USA; 4 Dalian Institute of Physics and Chemistry, Academy of China, Dalian, 116023, China

## Abstract

Core–shell structured catalysts, made by placing either a monolayer or a thin layer of a noble metal on relatively cheap core-metal nanoparticles, are fascinating and promising fuel cell catalysts due to their high utilization of noble metals. Here, we report our development of a core–shell structured catalyst, Ru@Pt/C, generated by a novel and facile pulse electrochemical deposition (PED) approach. We demonstrate that compared with a commercial Pt/C catalyst, this novel catalyst achieves over four times higher mass activity towards the anodic oxidation of methanol, and 3.6 times higher mass activity towards the cathodic reduction of oxygen. Importantly, we find that the intrinsic activity of Pt in this Ru@Pt/C catalyst is doubled due to the formation of the core–shell structure. The catalyst also shows superior stability: even after 2000 scans, it still retains up to 90% of the peak current. Our findings demonstrate that this novel PED approach is a promising method for preparing high-performance core–shell catalysts for fuel cell applications.

Low-platinum (Pt) catalysts, realized by either enhancing Pt utilization or reducing Pt loading and thereby decreasing its usage, have been one of the most interesting topics in proton exchange membrane fuel cell (PEMFC) research in the last decade^1^,^2^,^3^,^4^. PEMFCs are widely recognized as the most promising candidates for the next generation of clean power sources for electrical vehicles and other applications. However, major obstacles to the commercial application of PEMFCs are their use of Pt and their poor durability, both of which result in high cost. Recent breakthroughs in approaches to making core–shell structured catalysts fortunately may have shed some light on the route to achieving the commercial application of PEMFCs^5^,^6^,^7^. Placing a monolayer or a thin layer (i.e., composed of several atom layers) of Pt on a relatively cheap core-metal nanoparticle can result in high Pt dispersion, large active surface area, and high Pt utilization. In particular, the core–shell structure has emerged as a very attractive catalytic component, because unanticipated catalytic properties are often conferred upon the catalyst due to interactions between the shell and the core.

Adzic and co-workers^8^,^9^ first synthesized core–shell structured nanoparticles with a monolayer of Pt on a Pd core by using an underpotential deposition (UPD) method; a monolayer of Cu was deposited on the surface of Pd core particles, and this was followed by the galvanic exchange of Cu with a Pt salt solution to form a Pt monolayer. Scanning transmission electron microscopy (STEM) and X-ray absorption spectroscopy (XAS) confirmed the catalyst’s core–shell structure and the presence of a single monolayer of Pt^10^,^11^. This approach has been intensively investigated, and several core–shell catalysts with a Pt monolayer have been reported^12^. Compared with the core–shell structured catalysts prepared by other methods^13^,^14^,^15^, those prepared using the UPD method exhibited either much higher mass activity or much higher Pt utilization. In other words, UPD seems to be a superior method for the preparation of high-performance core–shell structured catalysts. However, UPD has some disadvantages: the high complexity of the process, as well as the large amount of inert gas needed to protect the system, hinder it from being used on a large scale. Furthermore, stability remains an issue for monolayer core–shell catalysts. Thus, there is a pressing need to explore new preparation methods.

A few researchers in the last decade have employed pulse electrochemical deposition^16^,^17^ to prepare fuel cell catalysts; however, this approach has received little attention because the catalysts generated by this method did not exhibit significant advantages, the particle size often was too large, and the size distribution was undesirable. Nevertheless, pulse deposition might be a good method for the preparation of shell layer of the core-shell structured catalyst, and further, that a few atoms shell layer on the core–shell catalyst might confer more stability than a monolayer. Based on these two ideas, we attempted to prepare core–shell catalysts with a shell of several atom layers by pulse electrochemical deposition, and we obtained exciting results. It should be mentioned that up to this point, we have not scoured the literature for research on core–shell catalysts prepared via this method.

## Results

### Preparation of Ru@Pt/C catalyst

**Figure 1:**
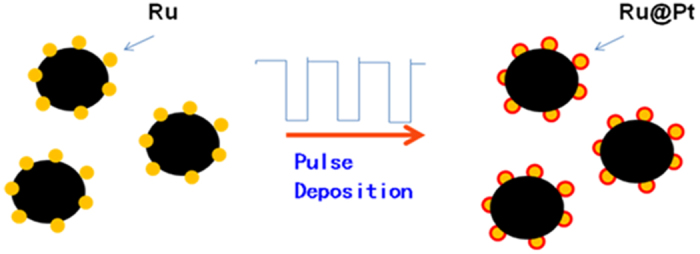
Synthesis method. Schematic diagram for preparing the Ru@Pt/C catalyst.

Carbon-supported (Cabot, USA) Ru nanoparticles, Ru/C, which had been prepared via a high-pressure colloidal approach, were used as the core substrate; the Ru content was 30 wt%, measured by thermogravimetric analysis (supplementary Fig. S6), and the particle size was ca. 2–3 nm. Using these Ru/C nanoparticles as the core, we prepared a core–shell structured catalyst with high Pt utilization using a pulse electrochemical deposition method in which an ultra-thin Pt shell is deposited on the surface of Ru nanoparticles supported by carbon black; we designate this catalyst Ru@Pt/C. Figure 1 illustrates the typical procedure we used for preparing our core–shell catalyst (see Method, below, for details). We selected Ru as the core metal based on the following considerations. Firstly, Ru is a much cheaper precious metal than Pt, its price being only one-tenth of the latter’s; secondly, the beneficial interaction between Ru and Pt may remarkably improve the catalyst’s performance. Indeed, as we expected, the catalyst showed amazingly high performance and Pt utilization.

### Characterization of Ru@Pt/C

We measured the deposited amount of Pt in our Ru@Pt/C catalyst by both the hydrogen underpotential desorption method (HUPD) and the atomic adsorption spectrum method (AAS). As shown in Table S1, the Pt content of the catalyst was 8.784 wt%, and the mass ratio of Ru to Pt was ca. 3.4:1.

**Figure 2:**
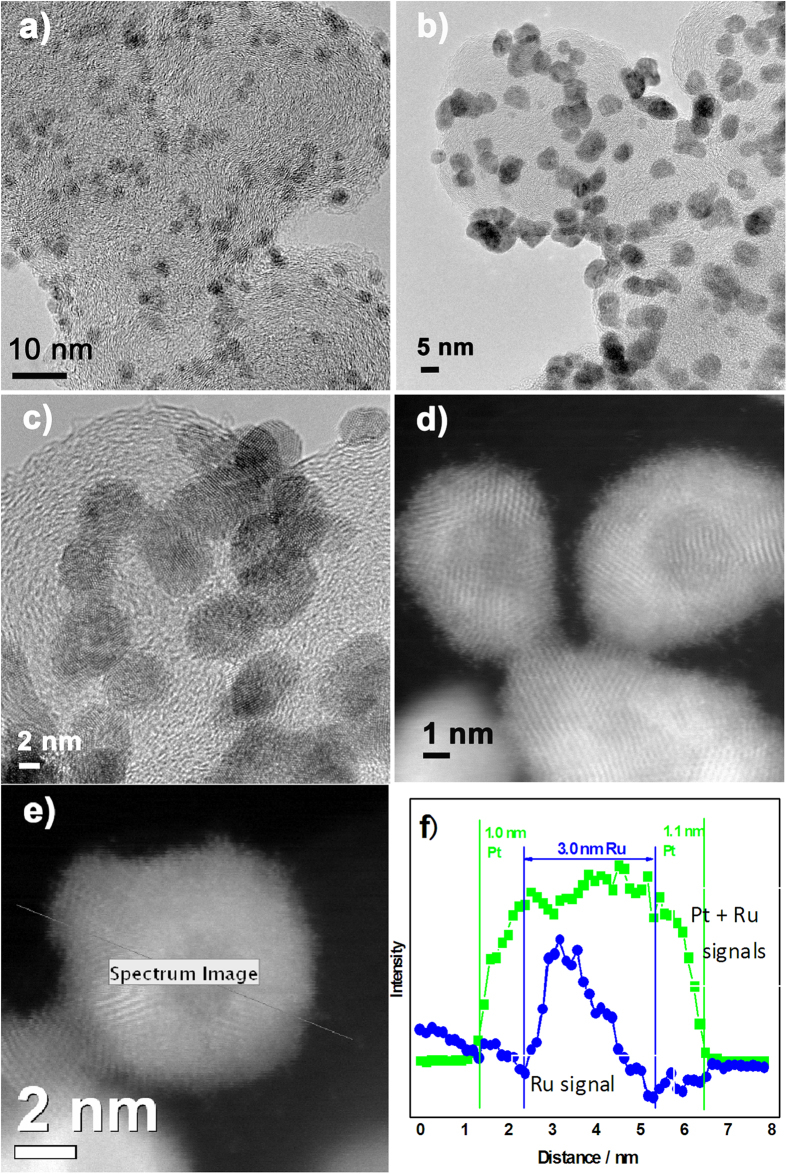
Microscopic and compositional observations of a single Pt monolayer on a Ru/C nanoparticle electrocatalyst. (**a**) HRTEM image of Ru/C; (**b** and **c**) HRTEM images of Ru@Pt/C; (**d**) HAADF-STEM image of Ru@Pt/C; (**e**) Ru@Pt/C nanoparticle chosen for EELS and ADF scanning analyses, and the scanning track line; (**f**) the Pt+Ru signal profile obtained from simultaneous ADF, and the Ru profile provided by STEM-EELS scanning analysis of Ru alone.

As shown in Fig. 2(a–c), images obtained by high-resolution transmission electron microscopy (HRTEM) revealed that the core–shell Ru@Pt nanoparticles were well dispersed on the surface of the carbon support. In addition, the particle size increased to ca. 4–5 nm (Fig. 2b), compared with the Ru nanoparticles (2–3 nm, Fig. 2a) that were used as the substrate for preparing the core–shell catalyst; this 1–2 nm increase in particle size confirms that a shell layer had formed, the thickness of which was ca. 0.5–1 nm, corresponding to 3–4 atom layers of Pt. Figure 2d presents a high-angle annular dark field (HAADF)/scanning transmission electron microscopy (STEM) image of Ru@Pt/C nanoparticles that very clearly reveals their core–shell structure. We also characterized an independent particle using STEM and EELS (electron energy-loss spectroscopy) linear scanning analysis. This particle and the scanning route are shown in Fig. 2e, while Fig. 2f shows the findings from the analysis: the green line in Fig. 2f shows the ADF signals obtained simultaneously with STEM-EELS, these being the sums of the signals from Pt and Ru; the blue line shows the EELS spectra of the Ru M_4,5_ edges. The results unequivocally indicate the core–shell structure of our catalyst: the Ru core is ca. 3 nm, the Ru@Pt is ca. 5 nm, and the shell layer is ca. 1 nm thick, implying that the shell may contain 3–4 atom layers of Pt.

**Figure 3:**
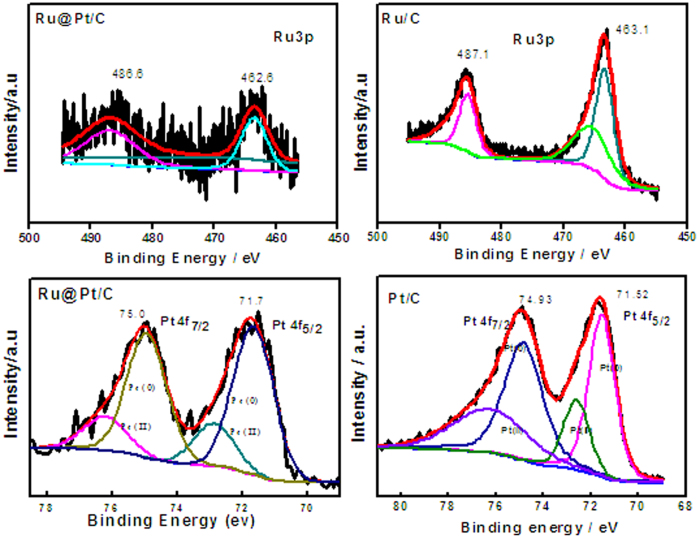
High-resolution XPS spectra of Pt and Ru in Ru@Pt/C, Ru/C, and JM-Pt/C.

We investigated the surface properties and the chemical states of Ru@Pt/C nanoparticles by X-ray photoelectron spectroscopy (XPS). This revealed no obvious change in the binding energy of Ru 3d in the Ru@Pt/C catalyst compared with the Ru/C substrate (Fig. 3); however, the binding energy of Ru 3p shifted positively by ca. 0.2–1.4 eV, indicating that Pt covered the Ru nanoparticles and that the Pt in the shell interacted with the Ru in the core. Furthermore, the binding energy of Pt 3d shifted negatively by ca. 0.2–0.3 eV with respect to a monometallic Pt sample, further confirming that charge transfer from Ru to Pt took place (due to platinum’s large electronegativity), and implying that an interaction occurred between Pt and Ru.

### Electrocatalytic performance

**Figure 4:**
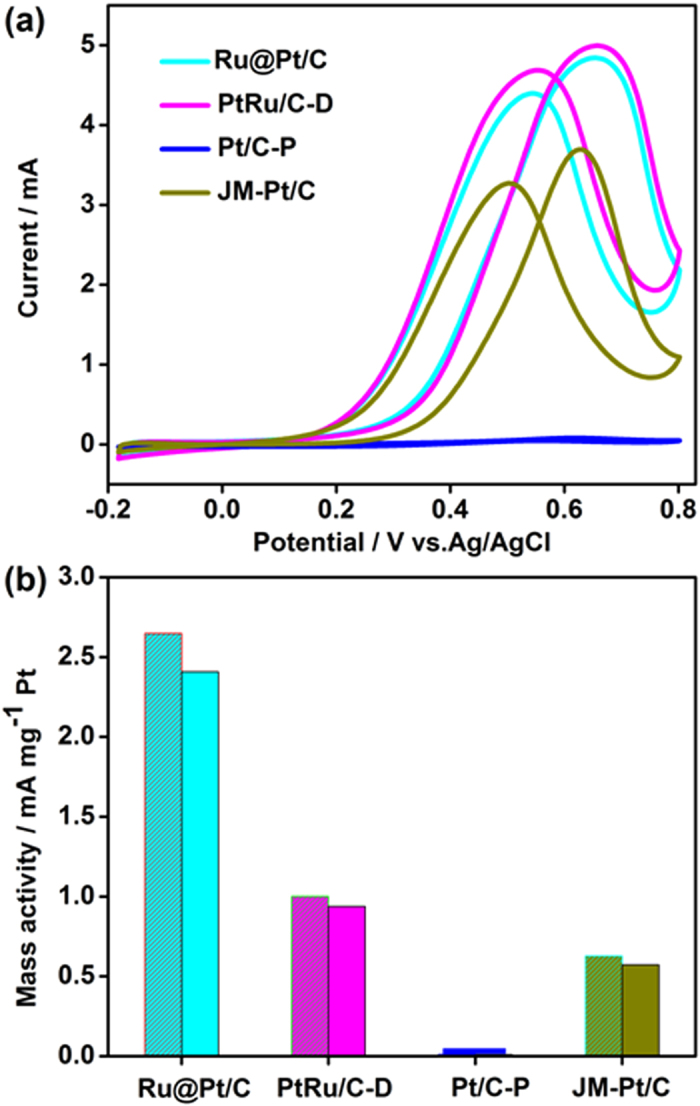
The methanol oxidation performance of Ru@Pt/C (8.798 wt% Pt), PtRu/C-D (25.34 wt% Pt), Pt/C-P (1.01 wt% Pt), and commercial JM-Pt/C (20 wt% Pt). (**a**) The original cyclic voltammograms in a solution of 0.1 M HClO_4_ and 1 M CH_3_OH, at room temperature and a scanning rate of 50 mV s^−1^. (**b**) The mass activity of Ru@Pt/C, PtRu/C-D, Pt/C-P, and JM-Pt/C towards the anodic oxidation of methanol; the shaded bars were calculated based on the forward peak current density, and the unshaded bars were based on the backward peak current density.

**Figure 5:**
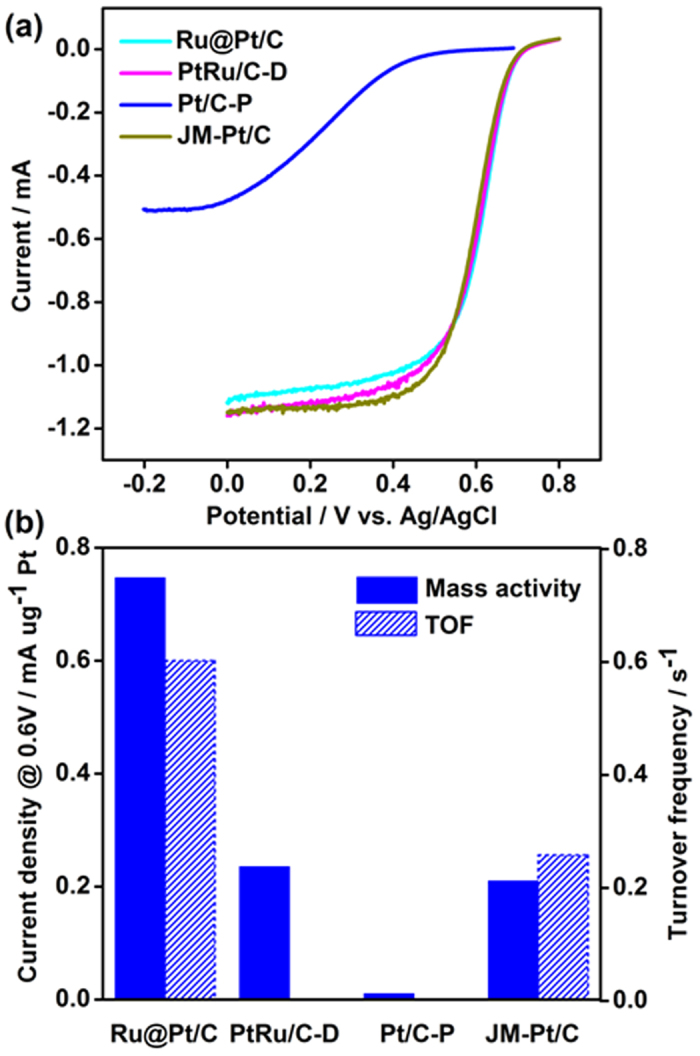
The ORR performance of Ru@Pt/C (8.798 wt% Pt), PtRu/C-D (25.34 wt% Pt), Pt/C-P (1.01 wt% Pt), and JM-Pt/C (20 wt% Pt). (**a**) The original oxygen reduction linear sweep voltammetry was recorded in O_2_-saturated 0.1 M HClO_4_ at room temperature, at a rotation speed of 1600 rpm and a scanning rate of 10 mV s^−1^. (**b**) The mass activity and turnover frequency values for Ru@Pt/C, PtRu/C-D, Pt/C-P, and JM-Pt/C towards the reduction of oxygen, calculated from the current density at 0.9 V (vs. RHE).

As shown in Figs 4 and 5, the core–shell catalyst Ru@Pt/C showed extremely high catalytic performance in the anodic oxidation of methanol and the cathodic reduction of oxygen.

For methanol oxidation, the mass activity of Pt reached 2.65 mA μg^−1^ Pt, over four times that of a commercial Johnson Matthey (JM) catalyst (JM-Pt/C, 0.627 mA μg^−1^ Pt; see Fig. 4b). Two factors might account for such high performance: the very high Pt dispersion greatly enhanced its utilization, and there was a synergetic effect between the Pt in the shell and the Ru in the core. Notably, when the core–shell structured catalyst was prepared by direct deposition (denoted as PtRu/C-D) instead of pulse deposition, its activity was only one-third that of Ru@Pt/C prepared via pulse deposition. For comparison, we also prepared a Pt/C-P catalyst by directly depositing Pt on Vulcan XC72R carbon using the same pulse deposition procedure; the mass activity of Pt in that case was only 3% that of Ru@Pt/C. Interestingly, oxygen reduction on Ru@Pt/C and the other catalysts demonstrated almost the same trend as the anodic oxidation of methanol. The mass activity of Pt in Ru@Pt/C was threefold that of JM-Pt/C and PtRu/C-D, and 100 times higher than that of Pt/C-P (Fig. 5b), implying that pulse deposition may play a crucial role in the high performance of Ru@Pt/C, along with the high dispersion of Pt that arises from the core–shell structure.

We suggest that the high performance of Ru@Pt/C prepared by pulse deposition may reflect the following. Firstly, the catalyst’s core–shell structure ensured high Pt dispersion on the surface of the core nanoparticles. The HRTEM and STEM results showed that Pt was deposited in an ultra-thin layer but had not formed independent Pt nanoparticles on the carbon support (Fig. 1). To help understand the roles of pulse deposition and of the Ru core, we prepared a Pt/C-P catalyst by directly depositing Pt on the carbon support using the pulse deposition method under the same conditions as for the preparation of Ru@Pt/C. Again, almost no Pt nanoparticles were found on the carbon support (Supplementary Fig. S1), confirming that high Pt dispersion can be realized via our method. However, the Pt loading in the Pt/C-P was only 1.01 wt%, and that catalyst exhibited very poor activity towards the anodic oxidation of methanol as well as the cathodic reduction of oxygen, confirming the importance of the Ru core and of having high Pt dispersion. The interaction between Pt and Ru, which the XPS measurements confirmed, is a second possible factor (Fig. 3). After the Pt shell was deposited on the Ru core, the binding energy of Pt 4f shifted by 0.1 to 0.2 eV, whilst that of Ru 3d shifted by 0.5 eV. Furthermore, the intensity of Ru 3d clearly decreased, which indicates the successful deposition of a Pt shell on the Ru core.

**Figure 6:**
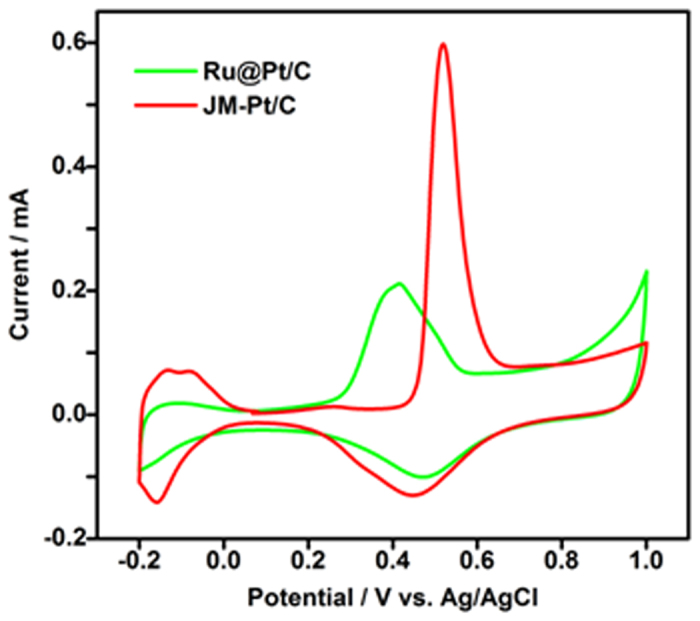
CO stripping voltammetry of Ru@Pt/C, PtRu/C-D, Pt/C-P, and JM-Pt/C after CO adsorption at −0.10 V vs. Ag/AgCl. During CO stripping, N_2_ aerated the working electrode compartment (flow rate: 100 mL min^−1^). Sweep rate: 20 mV s^−1^. Room temperature.

To further explore this interaction and its potential to improve catalytic performance, we used CO stripping method to measure the numbers of active Pt atoms on the JM-Pt/C and the core–shell Ru@Pt/C (Ru:Pt = 2:1) (Fig. 6). We then calculated the turnover frequency (TOF), based on the number of active Pt atoms. Interestingly, we found that the TOF (which is generally recognized as indicating a catalyst’s intrinsic activity) was more than double that of JM-Pt/C, revealing that the enhanced ORR performance of Ru@Pt/C resulted not only from the high Pt dispersion caused by the core–shell structure, but also from the enhanced intrinsic activity of Pt, which in turn may have been due to the interaction between Pt and Ru, as well as the quantum effects arising from high Pt dispersion. In other words, the Ru acts not only as a core element but also as a promoter of platinum’s intrinsic activity in the shell layer. With respect to Ru improving the intrinsic activity of Pt, we suggest that this may be caused by charge transfer from Ru to Pt, and by Ru eliminating poisonous intermediates that adsorb on the Pt sites.

The enhanced intrinsic activity of Pt in this Ru@Pt/C core–shell catalyst is a very important and valuable point, as is the fact that pulse deposition seems to produce quite different results from constant current deposition. To further understand the pulse deposition method, we prepared a RuPt/C “core–shell” catalyst using a constant current deposition method; we denote this as RuPt/C-D. The Pt loading of this catalyst was up to 25.34 wt%, and it exhibited poor performance. Its mass activity towards cathodic oxygen reduction and anodic methanol oxidation was only a quarter that of the Ru@Pt/C catalyst prepared via the pulse deposition method, although it was slightly higher than that of JM-Pt/C. From the HRTEM image (supplementary Fig. 2S), we can see that an enormous number of Pt nanoparticles, but not Pt layers, were deposited on the Ru/C, making it looks more dense with respect to the dispersion of Ru nanoparticles (see Fig. 2a). In conclusion, constant current deposition distributed the Pt not on the surface of the Ru nanoparticles but on the surface of the carbon support in Ru/C, implying the possibility that no core–shell structure can be generated via this method. This may be why no obvious mass activity improvement was observable for this catalyst. Whilst this finding is very interesting, we cannot as yet explain why pulse deposition can result in a core–shell structure; certainly, however, constant current deposition has not been similarly successful, up to this point. Further work is needed to reveal the mechanism by which pulse deposition induces the formation of a core–shell structure.

**Figure 7:**
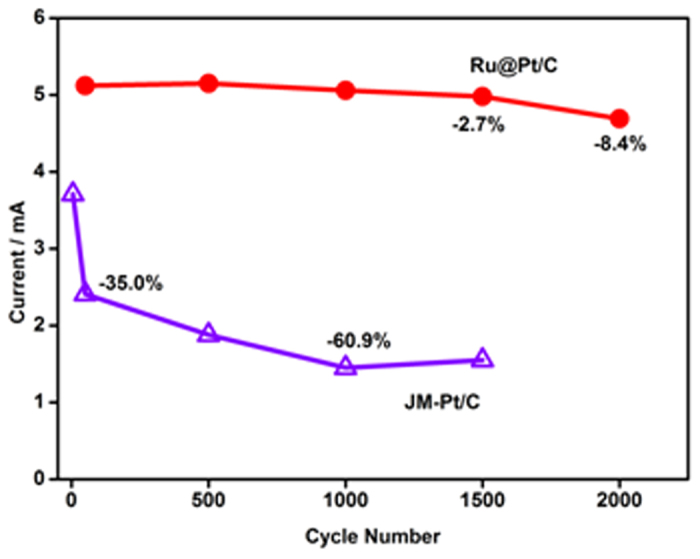
The relationship between the peak current of the anodic oxidation of methanol and the cycling numbers for Ru@Pt/C and JM-Pt/C. All data were recorded in a solution of 0.1 M HClO_4_ and 1 M CH_3_OH at room temperature and a scanning rate of 50 mV s^−1^.

Importantly, the Ru@Pt/C exhibited excellent stability towards the anodic oxidation of methanol. As shown in Fig. 7, after 2000 cycles, only a slight degradation of about 8.4% was observed. In comparison, the JM-Pt/C displayed significant degradation; beginning with the fifth scan, after 1000 cycles, the degradation reached 50%. In general, loss of catalytic activity after successive scans may result from decreased methanol concentration as it is consumed during cyclic voltammetry scanning. However, the loss may also be attributable to poisoning of and structural changes in the Pt nanoparticles, resulting from perturbation of the potential during scanning in an aqueous solution, especially in the presence of an organic compound. Sufficient improvement was noted in the Ru@Pt/C, with the anodic current decreasing slightly after only 1500 scans. The catalyst’s stability can be attributed to the synergetic effect between Ru and Pt, which essentially eliminated poisonous species on the electrode’s surface^18^,^19^,^20^. In contrast, the lesser stability of the commercial Pt/C catalyst may have resulted from the accumulation of CO adsorbed on its surface, because CO adsorbed on Pt is not easily oxidized or eliminated without the help of Ru.

## Discussion

We report here a new type of high-performance, core–shell structured catalyst, Ru@Pt/C, in which an ultra-thin Pt shell (3–4 atom layers) was deposited on Ru/C nanoparticles using a pulse electrodeposition approach. The catalyst exhibited three to four times higher activity towards the anodic oxidation of methanol and the cathodic reduction of oxygen than a commercial Pt/C catalyst. This finding implies that pulse deposition may result in ultra-high Pt dispersion and a strong interaction between the Pt shell and the Ru core, thus leading to high Pt utilization and excellent catalyst performance. Importantly, we found that the intrinsic activity of Pt in Ru@Pt/C was enhanced by a factor of 1.3 due to its core–shell structure. As well as high activity, the catalyst also showed ultra-high stability during both anodic methanol oxidation and cathodic oxygen reduction. These results might have been due to a synergetic effect arising from the co-existence of Pt and Ru. We believe that the ultra-high activity and stability of our Ru@Pt/C catalyst prepared by pulse electrodeposition make this an attractive approach for preparing core–shell structured catalysts.

## Methods

### Synthesis of Ru/C

Ru/C was prepared by an organic colloid method in an autoclave. 50 mg ruthenium(III) chloride hydrate was dissolved in acetone glycerol. Sodium citrate (with a molar ratio to Ru of 2.5:1) was then added under stirring until it dissolved completely. Next, we added 200 mg pretreated carbon black (Cabot Corp., S_BET_: 237 m^2^ g^−1^, denoted as C) whilst stirring, and then we adjusted the pH to over 10 by the drop-wise addition of 10 wt% KOH solution. We transferred this mixture into an autoclave with a Teflon liner, the temperature of which was held at 180 °C for 10 h. Next, we neutralized the mixture with 10 wt% nitric acid solution. The product was collected by filtration, washed thoroughly with deionized water, and dried at 70 °C under vacuum overnight.

### Synthesis of Ru@Pt/C

The prepared Ru/C typically was dispersed in a water/isopropanol/Nafion-ionomer solution to form an ink. Specifically, we prepared a solution of 77% isopropanol, 23% deionized water, and 0.15% Nafion ionomer by mixing 38.5 mL isopropanol with 11.5 mL deionized water and 1.5 mL 5 wt% Nafion-ionomer solution (Ion Power, Liquion 1100) in a 50 mL volumetric flask. Successively, 5 mg Ru/C and 2 mL water/isopropanol/Nafion-ionomer solution were measured into a 10 mL borosilicate vial then mixed thoroughly by sonicating for ≥60 minutes in a water bath (temperature <40 °C).

Next, a 5 μL droplet of the well-dispersed ink was pipetted onto a clean, polished, glassy carbon electrode (GCE, 5 mm diameter, Pine Research Instrumentation, USA), such that it completely covered the GCE but did not cover any of the Teflon. The ink droplet was dried under a weak infrared lamp to yield a smooth film that covered the entire surface of the GCE.

Finally, pulse electrochemical deposition of the Pt shell was carried out at room temperature using a three-electrode system with a Metrohm Autolab electrochemistry station (Model PGSTAT302N). A 3 M Ag/AgCl electrode was used as the reference and Pt gauze as the counter electrode. Before Pt deposition, the working electrode was reduced in a 1 M HClO_4_ solution that first was degassed with high-purity nitrogen. The working electrode was then cycled between open circuit voltage and −0.2 V until the oxidizing materials on it had been completely deoxidized. At the end of cycling, the working electrode was suspended at −0.2 V for 2 or 4 minutes, then removed to an electrolyte solution containing 0.5 M Pt(NH_3_)_4_Cl_2_/H_2_O, 0.1 M Na_2_SO_4_, and 1.25 M sodium citrate, which first had been degassed with high-purity nitrogen for 20 minutes. We then implemented pulse deposition by applying a pulsed current (3 mA cm^−2^) to the electrode; the turn-on time and turn-off time, respectively, were 0.0003 s and 0.00015 s.

## Additional Information

**How to cite this article**: Chen, D. *et al.* Ultra-high-performance core-shell structured Ru@Pt/C catalyst prepared by a facile pulse electrochemical deposition method. *Sci. Rep.* **5**, 11604; doi: 10.1038/srep11604 (2015).

## Supplementary Material

Supplementary Information
