# Supplementary material for: Ultra-high-performance core–shell structured Ru@Pt/C catalyst prepared by a facile pulse electrochemical deposition method
Source: Sci Rep. 2015 Aug 3;5:11604. doi: 10.1038/srep11604 (PMC4522673; doi:10.1038/srep11604)
Supplement: Supplementary Information [file srep11604-s1.pdf]

Supporting Information

Ultra-high performance core-shell structured Ru@Pt/C catalyst prepared by facile pulse electrochemical-deposition method

Dan Chen[†], Yuexia Li [†,‡], Shijun Liao[*,†], Dong Su[§], Huiyu Song[†], Yingwei Li[†], Lijun Yang[†], and Can Li[¶]

[†]Key Laboratory of Fuel-cell Technology of Guangdong Province, School of Chemistry and Chemical Engineering, South China University of Technology, Guangzhou, 510641, China,

[‡]Department of Chemistry, Datong University, Datong, 037009, China,

[§]Functional Nanomaterials Center, Brookhaven National Laboratory, Upton, NY 11973, USA,

[¶]Dalian Institute of Physics and Chemistry, Academy of China, Dalian, 116023, China.

Email: chsjliao@scut.edu.cn

1. **HRTEM and STEM images**

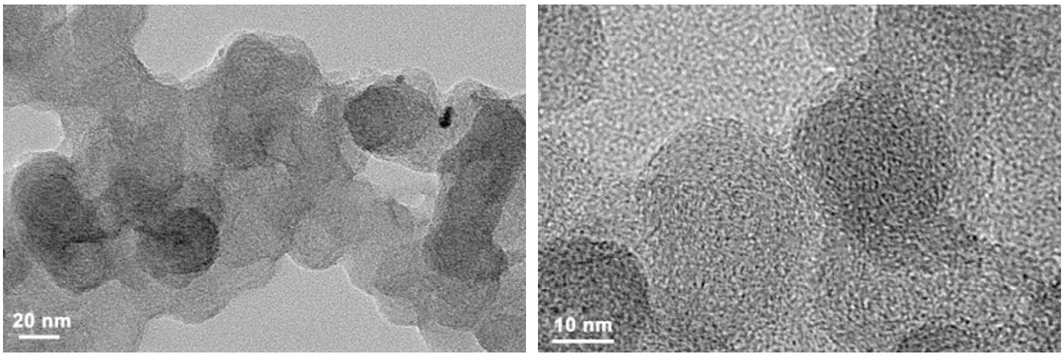

Figure S1. HRTEM images of Pt/C-P catalyst, which is prepared by depositing Pt on the carbon directly with pulse electrochemical deposition method.

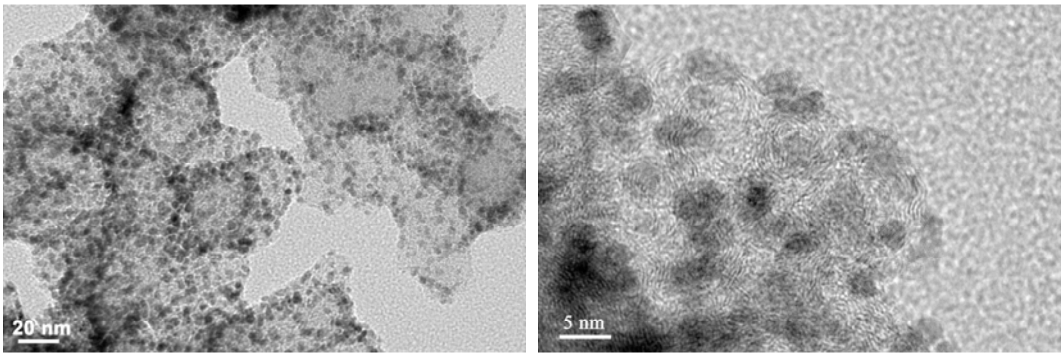

Figure S2. HRTEM images of PtRu/C-D catalyst prepared by depositing Pt shell layer on the Ru nanoparticles with constant current electrochemical deposition method, a lot of Pt was deposited.

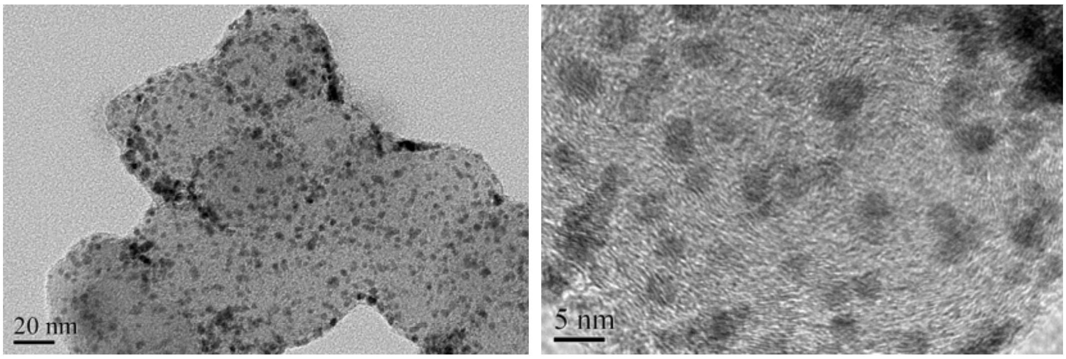

Figure S3. HRTEM images of Ru@Pt/C catalyst prepared by depositing Pt shell layer on the Ru nanoparticles with a pulse electrochemical deposition method.

2. Performance

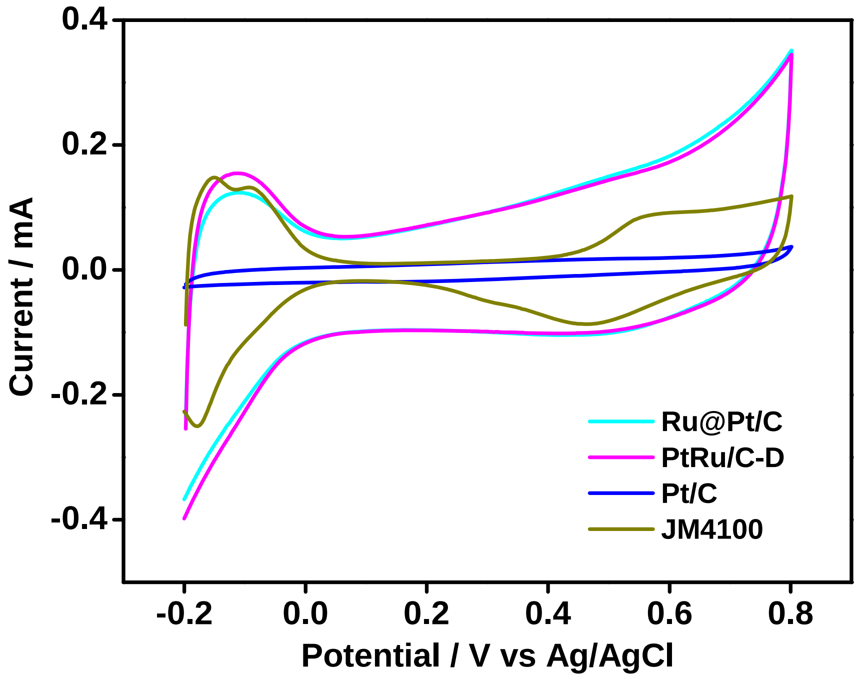

Figure S4. CV curves of various catalysts in 0.1 M $HClO_4$ solution at room temperature, the sweeping rate is 50 mV s^{-1}.

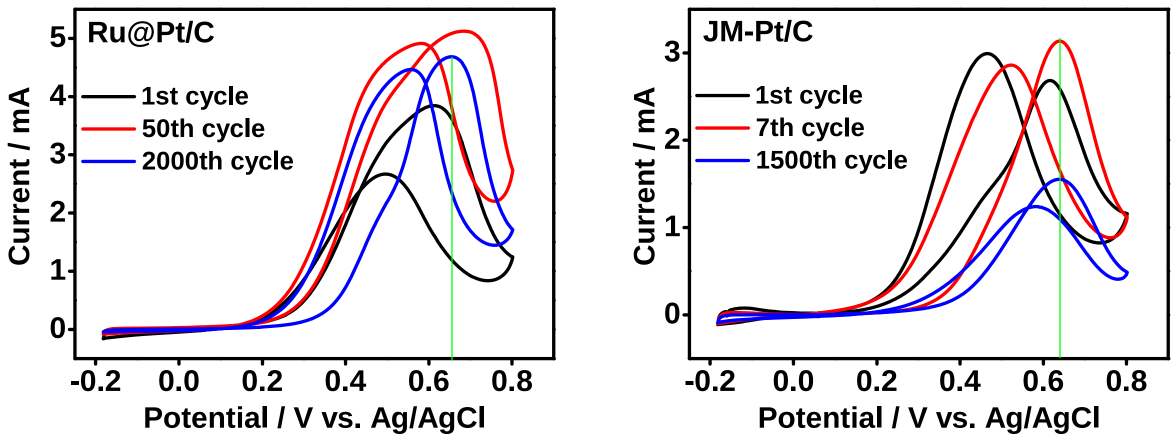

Figure S5. CV curves of 1th, 50th and 2000th cycles for the Ru@Pt/C catalyst, and CV curves of 1th, 7th and 1500th cycles for JM-Pt/C catalyst in 0.1 M $HClO_4$ and 1M CH_3OH solution at room temperature.

3. TG of Ru/C

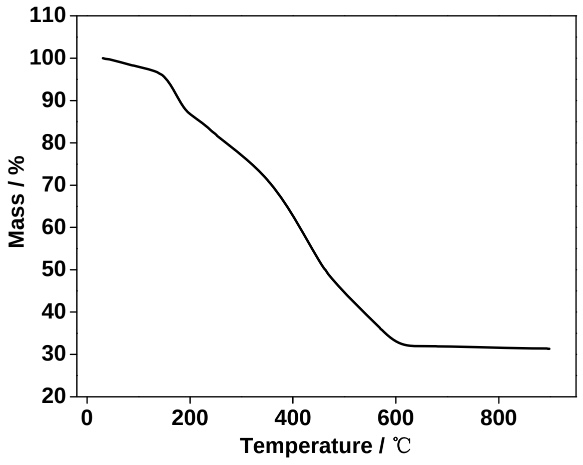

Figure S6. TG curve for Ru/C sample with Ru content of 30% by weight.

4. Pt content of catalysts

Table S1. The Pt content determined by Induction –Coupled Plasma AAS.

sample	Ru@Pt/C	Ru@Pt/C-D	Pt/C-P
Pt content / wt.%	8.798	25.34	1.01

5. K-L plots

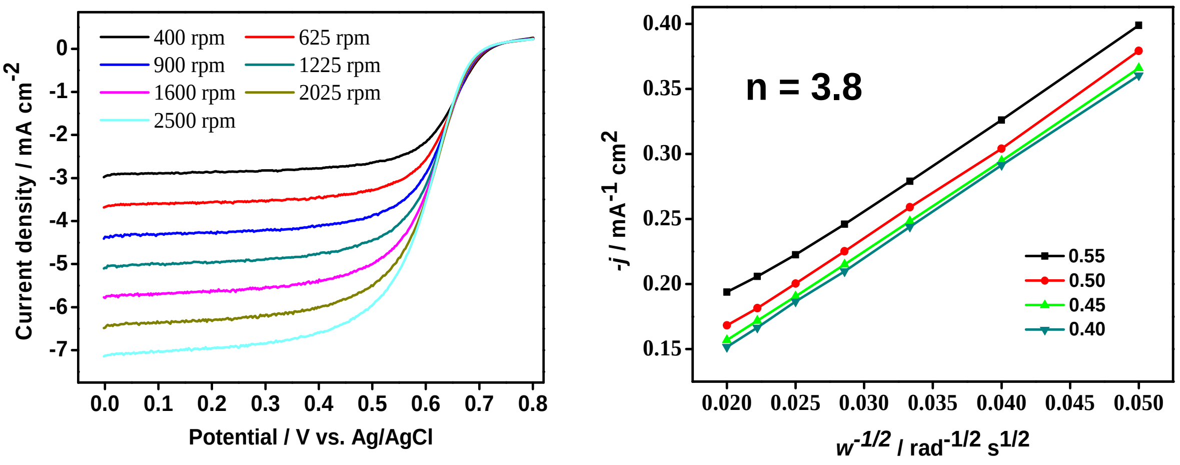

Figure S7. ORR polarization curves of Ru@Pt/C catalyst in O_2-saturated 0.1M $HClO_4$ solution at arious rotating speeds, and corresponding Koutecky-Levich plots.
